# Surviving starvation: essential role of the ghrelin-growth hormone axis

**DOI:** 10.1186/1753-6561-6-S3-O7

**Published:** 2012-06-01

**Authors:** Joseph L Goldstein, Tongjin Zhao, Daniel Sherbet, Michael S Brown

**Affiliations:** 1 University of Texas Southwestern Medical Center. Dallas, TX 75390, USA

## 

Chronic starvation is a repeated threat to survival of animals of all species. Indeed, about 15% of the current human population is estimated to suffer from severe malnutrition, and one-half of all deaths in children less than 5 years of age (~6 million deaths per year) arise from malnutrition. Over the centuries, starvation has exerted profound evolutionary pressure that has selected for a variety of adaptive mechanisms to support life. Paramount among these mechanisms is the necessity to maintain blood sugar concentrations sufficient for brain function. The adaptive mechanisms are particularly strained when chronic starvation has depleted the body of its other source of energy – namely, fatty acids stored as triglycerides.

Recently, our laboratory has begun to study the adaptation to chronic starvation, focusing on the essential roles of two peptide hormones, ghrelin and growth hormone. Ghrelin, a peptide hormone secreted by neuroendocrine cells in the stomach, was identified in 1999 by Kojima and Kangawa by its ability to stimulate release of growth hormone. In rodents and humans, plasma ghrelin rises before meals and declines after eating. Administration of excess ghrelin increases food intake, but knockout mice lacking ghrelin or its receptor have normal weight. Therefore, the true function of ghrelin has been enigmatic. Ghrelin is unique in that it requires a covalently attached 8-carbon fatty acid for activity, a modification conserved in all vertebrates. We identified ghrelin *O*-acyltransferase (GOAT), the enzyme that attaches octanoate to ghrelin. GOAT knockout mice cannot produce active ghrelin. Like ghrelin knockouts, GOAT knockouts have normal body weight. When these knockout mice are placed on a 60% calorie-restricted diet for 8 days, they are unable to maintain normal blood glucose and die. Restoration of ghrelin or growth hormone prevents death. Thus, ghrelin maintains blood glucose under conditions of severe calorie restriction, a function essential for survival in chronically starved rodents. Recent data on the mechanism by which the ghrelin-growth hormone axis preserves blood glucose will be presented.

